# Conducting and interpreting disproportionality analyses derived from spontaneous reporting systems

**DOI:** 10.3389/fdsfr.2023.1323057

**Published:** 2024-01-26

**Authors:** Paola Maria Cutroneo, Daniele Sartori, Marco Tuccori, Salvatore Crisafulli, Vera Battini, Carla Carnovale, Concetta Rafaniello, Annalisa Capuano, Elisabetta Poluzzi, Ugo Moretti, Emanuel Raschi

**Affiliations:** ^1^ Sicily Pharmacovigilance Regional Centre, University Hospital of Messina, Messina, Italy; ^2^ Uppsala Monitoring Centre, Uppsala, Sweden; ^3^ Unit of Adverse Drug Reaction Monitoring, University Hospital of Pisa, Pisa, Italy; ^4^ Department of Medicine, University of Verona, Verona, Italy; ^5^ Department of Biomedical and Clinical Sciences, Pharmacovigilance & Clinical Research, International Centre for Pesticides and Health Risk Prevention, ASST Fatebenefratelli-Sacco, Università degli Studi di Milano, Milan, Italy; ^6^ Department of Experimental Medicine, Section of Pharmacology “L. Donatelli”, University of Campania “Luigi Vanvitelli”, Napoli, Italy; ^7^ Department of Medical and Surgical Sciences, Pharmacology Unit, Alma Mater Studiorum, University of Bologna, Bologna, Italy

**Keywords:** disproportionality analyses, disproportionality, signal detection, SDR, adverse drug reactions, spontaneous reporting database

## Abstract

Spontaneous reporting systems remain pivotal for post-marketing surveillance and disproportionality analysis (DA) represents a recognized approach for early signal detection. Although DAs cannot be used *per se* as a standalone approach to assess a drug-related risk and cannot replace clinical judgment in the individual patient, their role remain irreplaceable for rapid detection of rare and unpredictable adverse drug reactions with strong drug-attributable component (e.g., designated medical events), especially when developed by a multidisciplinary team and combined with a careful case-by-case analysis (individual inspection of reports for causality assessment or to uncover reporting patterns and clinical features). In the recent past, a remarkable increase in publications of pharmacovigilance studies using DAs was observed, albeit the quality was debated: several publications contained “spin”, namely, misinterpretation of results to infer causality, calculate incidence, or provide risk stratification, which may ultimately result in unjustified alarm. The development of dedicated Guidelines by the international READUS-PV project (https://readus-statement.org/) will allow reproducible and transparent publication of accurate DAs, thus supporting their real transferability and exploitation by regulators and clinicians. This review offered a perspective on methodological aspects (and understanding) of DAs, their rationale, design, reporting, and interpretation.

## 1 Background

Disproportionality analysis (DA) in spontaneous reporting databases of adverse drug reactions (ADRs) refers to validated quantitative methods used for signal detection in pharmacovigilance. Box 1 summarizes the most frequently used measures of disproportionality and the formulae for their calculation. DA methods allow to detect higher reporting frequencies of specific drug‐event combinations in comparison with general reporting frequencies detected in a reference dataset which usually consist of the overall database (Bate and Evans, 2009; Hauben and Bate, 2009; Candore et al., 2015; European Medicine Agency, 2017). What is considered statistically disproportionate is determined by what might be expected by chance and, in case of spontaneous reporting databases, it could be derived from the proportionate representation of all of the reported drug-event combinations in the entire database (ICH. International, 2023). For instance, frequentist DA methods (i.e., proportional reporting ratio—PRR; reporting odds ratio—ROR), based on a 2 × 2 contingency table (Box 1), relate the observed count of a drug-event combination with an “expected value” extrapolated from the counts of all other drugs and adverse events (AEs) in the database (full data reference set as primary reference group) (Evans et al., 2001; Rothman et al., 2004). An example of Bayesian method is the Information component (IC) which is defined as the logarithm of the ratio of the observed rate of a specific AE to the expected rate of AE under the null hypothesis of no drug-AE association (Box 1) (Bate et al., 1998). When correctly interpreted, these measures can be very useful for generating hypotheses about unknown adverse drug reactions warranting further investigation to be validated as safety signals (Bate and Evans, 2009; Hauben and Aronson, 2009). However, DA measures cannot estimate risks or necessarily account for a causal association but only facilitate the identification of AE supposed to have a higher-than-expected reporting frequency.

Box 1 **Most frequently used disproportionality methods**

Frequentist methods

**Proportional Reporting Ratio (PRR)** (Evans et al., 2001)**, Reporting Odds Ratio (ROR)** (Rothman et al., 2004)

These methods are based on a 2 x 2 contingency table that relates the observed number of a drug-AE combination of interest to all other drugs and events in the database, which together constitute the background.

**Table:** 

	Adverse event of interest (Y)	Other adverse events	Total
**Using drug of interest (X)**	*A*	*B*	(a+b)
**Using other drugs**	*C*	*D*	(c+d)
**Total**	(a+c)	(b+d)	(a+b+c+d)

When examining a combination of drug (X) and adverse event (Y) combination in spontaneously reported data a ‘two by two’ contingency table can be created, including the numbers of reports for the combination of interest. The first row contains the observed number of reports of drug X and adverse event Y of interest (“a”); the next displays the number of reports of other adverse events attributed to drug X (“b”, except the event of interest); the second row represents the number of reports describing the AE of interest Y for all other drugs in the database (“c”, except the drug X); lastly, “d” stands for all other reports attributed to all other medications and other AEs in the database. The conditions for detecting a signal of disproportionate reporting (SDR) are defined below.

$$\text{ROR}=\frac{a/c}{b/d}$$

Statistical threshold based on 95% confidence interval (CI)

– Lower bound 95% CI >1; – Number of reports ≥3 (or ≥5)*

$$\text{PRR}=\frac{a/\left(a+b\right)}{c/\left(c+d\right)}$$

1. Using 95% confidence interval (95% CI)

– Lower bound 95% CI >1; – Number of reports ≥3* (or ≥5)*

2. Using chi-square statistic (with Yates’s correction)

– PRR ≥2; – Chi-square ≥4; – Number of reports ≥3

* These thresholds are most frequently used, although the minimum number of cases can be modified depending on different aspects, including the database and the drug/event under investigation (Slattery et al., 2013)

Bayesian statistics

**Information Component (IC)** (Bate et al., 1998; Norén et al., 2013)

IC represents a measure of disproportionality that compares the observed and the expected reporting of a drug‐AE combination. The IC value is calculated using a logarithmic scale, with a value of zero indicating that the observed number of reports for a drug-ADR pair is equal to the expected rate of reporting for all drug-event combinations in the database. A positive IC value, that usually requires that the lower 95% CI of the IC exceed zero, indicates that a specific drug‐AE combination is reported more frequently than expected, suggesting a safety signal. The higher the IC value, the more prominently the combination distinguished itself from the background. The IC is given by the following expression:

$$\text{IC}=\log2\frac{a\;\left(a+b+c+d\right)}{\left(a+c\right)\left(a+b\right)}$$

IC025 = lower limit 95% confidence interval. If the IC value increases over time, and the IC025 value is positive, this suggests a correlation between the drug and the AE.

**Multi-item Gamma-Poisson-Shrinker (MGPS) Algorithm** (Szarfman et al., 2002; Gould, 2003; Hauben and Bate, 2009)

The Multi-item Gamma-Poisson-Shrinker (MGPS) algorithm is an empirical Bayesian method that matches the observed and the expected counts of specific drug‐event combinations.

The MGPS algorithm produces adjusted relative reporting ratios, also referred to as Empirical Bayes Geometric Mean (EBGM) values. For each drug-event combination in the database, a Relative Reporting Ratio (RR) is defined as the observed count (N) divided by the expected count (E), N/E, for any adverse event associated with a specific drug in comparison to all other drugs and adverse events in the entire database. The expected counts (E) are computed using a stratified, full-independence model. This model aids in identifying potential drug-event associations that deviate significantly from what would be expected under the assumption of no association.

Furthermore, the MGPS calculates two-sided 90% confidence intervals (EB05 and EB95) for these EBGM scores. EB05 greater than or equal to 2 is used as a threshold in data mining to identify signals.

DA findings requires cautious interpretation, assessments of the risk of bias, and consideration for alternative explanations other than causal association between the drug and the AE (Mouffak et al., 2021). Indeed, clinical assessment (qualitative analysis) remains essential before drawing any causal inference from DA measures. Consequently, DA are insufficient to confirm safety signals and rarely support regulatory decisions on their own. In this regard, several published reviews and some official documents issued by regulatory agencies proposed key requirements for spontaneous reporting analyses (ENCePP, 2013; Wisniewski et al., 2016; European Medicine Agency, 2017).

Despite these premises, a growing literature based on disproportionality methods claims to identify and confirm associations between a given drug and a given adverse reaction, by presenting all the DA results without adequate clinical evaluation for confirmation (Sartori et al., 2023). Most of these studies derive from open-access pharmacovigilance databases and the relevant public dashboard, characterized by significant restrictions in performing both quantitative and qualitative analyses (Fouretier et al., 2016). Their results could often be misinterpreted because of spins, such as the use of causal language or the inappropriate extrapolation of definite safety risks referring to DA findings taken alone (Mouffak et al., 2021). This tendency of inappropriately using and interpreting spontaneous reporting data and DAs to make claims on causal inference or estimate about the incidence or prevalence of ADRs, and to compare the safety profiles of different drugs has been called “pharmacovigilance syndrome” (Greenblatt, 2015).

In the light of this phenomenon, this narrative review is aimed at: a) clarifying the meaning of signal and DA; b) illustrating the role and positioning of DA in the signal management process; c) illustrating elements of signal validation that are essential integration of measure of disproportionality; d) describing the rationale and the possible research questions that DA can address; e) discussing the possible interpretations and implications of statistically-significant disproportionality; and f) identifying some contexts where DA may have been used inappropriately or was insufficient to assess drug safety issues.

This review relies on research experience of the authors (Raschi et al., 2019a; Sultana et al., 2019) and should not be intended as systematic, but rather represents the result of recent scientific debate among members of the Italian Chapter of the International Society of Pharmacovigilance (https://isoponline.org/chapters/italy/).

## 2 Signals, signals of disproportionate reporting, and risks

In 2009 the Council for International Organizations of Medical Sciences adopted Hauben and Aronson’s definition of a “signal of suspected causality” as: “Information that arises from one or multiple sources (including observations and experiments), which suggests a new potentially causal association, or a new aspect of a known association, between an intervention and an event or set of related events, either adverse or beneficial, which would command regulatory, societal or clinical attention, and is judged to be of sufficient likelihood to justify verificatory and, when necessary, remedial actions” (Hauben and Aronson, 2009).

The same authors (Hauben and Aronson, 2009) further distinguish “signal of suspected causality,” or more easily “signal”, from “signal of disproportionate reporting” (SDR), defining the latter as “numerical output of disproportionality analysis.” Having drawn the distinction between signals and SDRs, in the interest of appropriate use of terminology, it is indispensable (Raschi et al., 2016a) the concept of SDR be disentangled from that of “risk of harm,” that is: “the combination of the probability of occurrence of harm and the severity of that harm” (ICH. International, 2023). SDRs are probabilistic computations of reporting frequencies (observed vs. expected ratios) that quantify the likelihood of reporting suspected ADRs to a database. On the other hand, risks of harm are probabilistic estimates of the occurrence of one or more ADRs in a target population. Although few studies showed a weak correlation between SDRs and relative risk for known association, results from DA, even when conducted appropriately, cannot automatically translate to actual risk of harm (see Section 7.1) (Maciá-Martínez et al., 2016; Beau-Lejdstrom et al., 2019; Gatti et al., 2021a; Khouri et al., 2021a). Beyond terminology alone, it is imperative to refer to any findings from DAs as SDRs, and not as “risks,” to favor transparency of reporting results and avoid the so-called *spin* (Mouffak et al., 2021). SDRs and signals fit within the frame of signal management, i.e., the set of activities performed to determine whether there are new risks of harm, or whether there are new aspects of known risks of harm, associated with a medicinal product (European Medicine Agency, 2017).

## 3 Position of signal detection and signal validation in the signal management process: the role of signals of disproportionate reporting

Disproportionality analysis is typically employed in the earliest stage of signal management in databases of reports of suspected ADRs (signal detection). Whether applied to large or small databases (≥500 reports) (Caster et al., 2020), DA will give rise to false positives, the rate of which depends on the methods used (Harpaz et al., 2017; Pham et al., 2019) and the pre-established thresholds that define an SDR (Candore et al., 2015). Although there are means to curtail the rate of false positives, such as sensitivity analyses including background (Grundmark et al., 2014) or subgrouping/stratification (Hopstadius et al., 2008), the high resources required to follow-up all detected SDRs (Alvarez et al., 2010) solidify the role of DA as a first-pass screening method.

Signal validation—subsequent to signal detection—is the step of the process ensuring that a) the information on the suspected ADR is novel; b) the statistical findings hold when accounting for the strength of the evidence in the reports—among other aspects (European Medicine Agency, 2017). In practice, signal validation entails verifying whether SDRs are included in regulatory documents (e.g., risk management plans, periodic safety update reports) or reference materials (e.g., Summaries of Products Characteristics, SmPCs). Most importantly, signal validation requires a clinical review of the reports that, through the detection phase, numerically supported the SDRs. Such a review is concerned with ensuring that the data are of sufficient quality to allow for signal assessment, that the reports share similar clinical characteristics, or, more broadly, that they suggest causality according to, for example, the viewpoints of Bradford Hill (HILL, 1965; European Medicine Agency, 2017). In summary, to make the best use of available resources, detected SDRs require clinical review of the reports (Wisniewski et al., 2016). Examples of works that combine DA with a clinical review of reports may be found (de Jong et al., 2012; Tarapués et al., 2013; Viola et al., 2014; Béné et al., 2016; Valdiserra et al., 2023).

**FIGURE 1:**
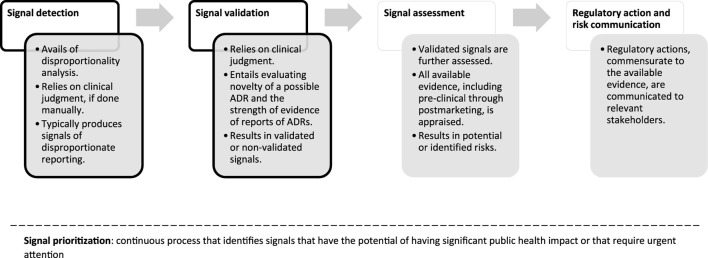
Flowchart of signal management, simplified and applied to reports of suspected ADRs. For a complete overview of the process, please see Good Vigilance Practices Module IX (European Medicine Agency, 2017). Throughout the phases of signal management, signals are continuously prioritized within signal prioritization. Shape borders in black indicate the steps that are most relevant to signals of disproportionate reporting.

In Figure 1, we report a schematic representation of the process of signal management and the position of signal detection and signal validation steps. In the next paragraphs we discuss in detail two essential steps of signal management that pertain SDRs that the researchers might consider and discuss, namely, novelty and strength of evidence.

## 4 Integrating measures of disproportionality with clinical and pharmacological assessments

### 4.1 Checking the novelty of the suspected adverse drug reaction

As mentioned above, the novelty of a suspected ADR should be checked after the detection of an SDR. Again, from the definition of signal, one should be concerned with the suggestion of a new potentially causal association or new aspects of a known one. The reader might rightfully ask: “*New to whom?*”.

Healthcare professionals, researchers, or regulators might find an ADR to be new: healthcare professionals may have never encountered it in their practice; researchers might detect an SDR for the first time in a database; and regulators might not have included the ADR in relevant SmPCs. In European guidelines, novelty of an ADR refers to the latter kind of scenario. Practically speaking, ensuring that a detected association is new involves checking its presence in SmPCs or critically reviewing these documents (Lindquist et al., 2000; Scholl and van Puijenbroek, 2012; Raschi et al., 2015). The underlying assumption of this approach is that regulatory documents are evidence-based, are used as reference materials in clinical practice, and have legal value. Notably, only the last statement is true: indeed, a number of ADRs are listed in the SmPCs only cautiously, without actual evidence of causal association, and health professionals rarely use SmPC as reference for their clinical decisions (McMahon and Preskorn, 2014).

Nevertheless, the SmPC currently still represents an objective and easy-to-access reference for assessing the novelty of a signal. Another barrier to the use of SmPC is the lack of established methods to determine whether detected associations match or diverge from the contents of SmPCs. As these supports include ADRs or AEs coded to the Medical Dictionary for Regulatory Activities (MedDRA), an automatic approach might be able to verify that the MedDRA term under study is present or not from the SmPCs. However, compared to its use in signal detection MedDRA is going to be applied conceptually differently in SmPCs; for healthcare professionals to make the best use of them, clinically related terms are going to be encapsulated in single expressions: the MedDRA Labelling Groupings (Große-Michaelis et al., 2023). One such example might be the MedDRA Preferred Terms “acute kidney injury” and “subacute kidney injury” subsumed under “acute kidney injury” in an SmPC. Moreover, SmPCs contain inconsistencies at a national level, across products in different (Sawalha et al., 2008; Al-Aqeel, 2012) or the same (Savill and Bushe, 2012) therapeutic class, or at an international one (Alloza and Lasagna, 1983; Pfistermeister et al., 2013; Lee et al., 2014; Noh et al., 2018). These inconsistencies also extend to expressions relating to drug-drug interactions (Jeong et al., 2019), or even to comparisons between generics vs. reference products (Sillo et al., 2018). Thus, the mere absence of a MedDRA term from the SmPC might not necessarily engender novelty. Instead, clinical reasoning ought to guide the assessment of novelty of an SDR, coupled with an appropriate choice of SmPCs to consult, which should account for their inconsistencies.

Considering the aforementioned challenges and limitations in the critical analysis of SmPCs, researchers should consider additional sources to check the novelty of an SDR, for instance by performing a structured literature review (e.g., an overview of systematic reviews) that require harder efforts, but may provide a more reliable support to the prioritization of SDR with likely clinical relevance (Raschi et al., 2019b).

### 4.2 Strength of evidence of the supporting reports

If an SDR has been deemed as novel, signal validation prescribes that the reports in its support be clinically reviewed. It would be beyond the scope of this work to provide a detailed guide on how to clinically review reports; readers should be mindful that there are no gold standards for these activities but the possible methods to assess causality in single or series of reports have been published elsewhere (Agbabiaka et al., 2008). Irrespective of methods used to appraise the reports, case narratives are an essential section to review. Not only case narratives have been weighed in semi-qualitative signal detection methods (Caster et al., 2014), but they have been found to provide information on medical history, and time course and diagnostic work-up of suspected ADRs (Szarfman et al., 2006; Klepper and Edwards, 2011). For reports submitted by patients, it was also possible to better understand changes to their quality of life (Inácio et al., 2018; Watson et al., 2018).

The necessity of a clinical review of the case narratives brings about an important aspect to consider when conducting DA, that is the availability of the data themselves. While the structured fields of raw exports of databases such as the Food and Drug Administration Adverse Events Reporting System (FAERS) are accessible online, narratives are sensitive information that require anonymization to ensure safekeeping of patients’ privacy and are not publicly available. Therefore, to carry out a thorough signal validation, it may be necessary to obtain narratives through Freedom of Information Act Requests, submitted to the competent regulatory agency or authority, when these data are unavailable to the researcher (US national Archives, 2023).

## 5 Rationale for conducting a disproportionality analysis

More than 10 years ago, the British Journal of Clinical Pharmacology opened the debate on the strengths and limitations of DAs, thus challenging, for the first time, the issue of publishing relevant results in medical journals (De Boer, 2011; Montastruc et al., 2011).

A key overlooked aspect is represented by the rationale and actual added value of a DA for the various stakeholders. In other words: “*which kinds of research questions can be addressed by a DA?*”. Of note, notwithstanding a remarkable increase in published DAs in recent years from academia (Sartori et al., 2023), most of them remains unnoticed by regulators and not corroborated by subsequent research (Dhodapkar et al., 2022). The availability of already published pharmacoepidemiological and/or spontaneous reporting data should be carefully taken into account before planning a DA, to avoid research waste and redundancies. In particular, the question arises on the actual added value of a newer but similar pharmacovigilance study using the same spontaneous reporting system. Reproducibility is vital in modern pharmacovigilance, especially when dealing with the FAERS archive, which requires extensive data processing and curation (see Section 6) before conducting a DA. In our opinion, replication studies are welcome, provided that they are carefully planned to offer an additional complementary perspective, for instance by providing a case-by-case analysis or by accounting for previously unrecognized biases to increase the robustness of a signal.

Moreover, the type of drugs and/or safety issues under investigation may drive the choice of the most appropriate pharmacovigilance data source. An appraisal of signals identified by the FDA from the FAERS concluded that DAs are effective for: 1) early detection of post-marketing risks with new drugs (namely, those with less than 5 years on the market), likely related to the so-called Weber effect; 2) continuous monitoring of old medications (due to prescribing changes, long‐term use); 3) identifying potentially overlooked non-serious events such as procedural complications (Fukazawa et al., 2018). With regard to the type of event, there is general agreement that spontaneous reporting systems represent the best source of data to investigate the so-called designated medical events (DMEs), namely, rare but serious side effects with strong drug-attributable component (e.g., Torsade de Pointes) that may escape detection from healthcare databases and pivotal trials (Harpaz et al., 2013; Raschi et al., 2016b).

Therefore, we believe that the key consolidated goal of a DA remains early detection of rare, unexpected, late-onset, or long-lasting ADRs (including those from drug interactions), which cannot be fully appreciated during the pre-marketing phase (Raschi et al., 2022). The “trigger” should be clinical practice, namely, case report/series, with plausible pharmacological basis. In specific areas, such as rare diseases, some specific regulatory aspects might support a stringent post-marketing monitoring of drugs receiving fast-track approval with preliminary benefit-risk evaluation or under specific risk management plan. This is especially the case of biologics/biotechnological drugs (Giezen et al., 2010; Cutroneo et al., 2014) and advanced therapy medicinal products with peculiar pharmacokinetics/pharmacodynamics (Fusaroli et al., 2022), for which mapping global safety profile through the agnostic/untargeted analysis may be warranted. These hypothesis-free studies aim to describe the entire spectrum of ADRs for a specific drug or drug class, or to explore all drugs responsible for a given ADR. A systematic review (Gaucher et al., 2023) of 92 pharmacovigilance studies (mostly employing DA as statistical approach) found that multiple testing was not adequately addressed in most studies, potentially leading to false positive signals, thus arguing for the establishment of specific guidelines. We also support specific criteria and case-by-case analysis to actually prioritize ADRs of special interest.

An emerging use of DA is represented by the combination with other source of data, including *in vitro/ex vivo* assays to corroborate the biological plausibility (Salem et al., 2019), or electronic healthcare records (EHRs) to substantiate (or refuse) the research hypothesis or, under certain circumstances, to enhance signal detection for events with high background incidence (Pacurariu et al., 2015; Roberto et al., 2023; Wang et al., 2023). The correlation between disproportionality measures and pharmacokinetic/pharmacodynamic features (e.g., lipophilicity, receptor affinity/occupancy) may be used to explore the underlying pharmacological basis. This is illustrated by a number of examples in the recent past, especially in the neuropsychiatric area (Mazhar et al., 2019; Gatti et al., 2021b; Fusaroli et al., 2023). Furthermore, the traditional analytical approaches to the analysis of adverse events can take advantage from the integration with evidence coming from genomics data, which can provide additional support to understand the biological plausibility as well as to describe the relationships between interacting components. As an example, the so-called “molecular expansion of adverse event data” is a promising approach to explore adverse events by providing mechanism-based context for the assessment of drug safety (Boland et al., 2016). In particular, the integration of DA with genomics data and molecular data concerning both drugs and biological targets allows for the improvement of the analysis of adverse event by providing insights that may be helpful to understand drug-target interactions within the context of a biological system. As such, the correlation between DA and both molecular features may lead to valuable implications for the assessment and the prediction of ADRs, as well as for precision medicine and routine clinical practice. Although the approach of coupling pharmacovigilance with pharmacometrics has attracted interest, there are still important methodological aspects that are not fully standardized, including the source of pharmacodynamic data (e.g., ChEMBL, DrugBank databases), relevant index (e.g., calculation of receptor occupancy) and management of missing/multiple data, as well as the statistical model to assess the target-event relationship. Moreover, this approach may not be suitable for drugs with unconventional or complex drug-target interaction (e.g., gene therapy) or immunological ADRs (Soldatos et al., 2022).

Another starting point for performing DAs may be simply represented by an event (e.g., rare diseases with unclear multi-factorial etiology) for which possible drug role in its occurrence should be investigated. By this approach, not only signals may arise, but also suggestions for mechanisms of disease and, even, for possible new pharmacological strategies. In particular, drugs arising from DAs should be further considered for their typical biological targets, by accessing literature and pharmacodynamic databases, since these targets could also represent a key-point for the specific disease development (Gaimari et al., 2022).

A mixed approach combining a meta-analysis of clinical trials with a DA might be a complementary strategy to comprehensively describe the spectrum and other clinical features of a given safety issue (Wang et al., 2018; Mouffak et al., 2021). Moreover, the analysis of social media and drug information databases may reveal the patient’s perspective on concepts (e.g., anxiety) overlooked/underexplored in traditional sources (Smith et al., 2018).

Finally, the investigation of methodological aspects represents an important goal of DA. While in the recent past the vast majority of efforts were directed in demonstrating/handling of reporting biases (Arnaud et al., 2016), in the current era of artificial intelligence ongoing challenges are represented by automation in detection, assessment and prioritization of safety signals through machine-learning algorithms and natural language processing (Ball and Dal Pan, 2022; Battini et al., 2023a; Al-Azzawi et al., 2023).

## 6 Possible interpretation of a disproportionality signal

A number of common pitfalls can be identified in presenting study results from DAs. Due to inherent limitations, causality, risk ranking, incidence, prevalence and reporting rate cannot be calculated or claimed (with the exception of vaccines if the background rates are known or in case of sufficient drug utilization data) (Raschi et al., 2018; Faillie, 2019; Cortes et al., 2023; Khouri et al., 2023). A recent meta-research on 100 randomly selected published studies noted a misinterpretation of results, also called “spin”: many DAs intentionally or unintentionally overstate the strength of causal links, lack proper handling and discussion of biases, or over-extrapolate results to provide clinical recommendations or compare drug safety profiles, notably in the abstracts (Mouffak et al., 2021).

An accurate discussion of SDRs is mandatory, to avoid two possible scenarios: a) overlooking a true safety problem or b) emphasizing the importance of a biased SDR (Bate and Evans, 2009; Raschi et al., 2018; Faillie, 2019). Therefore, alternative (not drug-related) causes should be excluded, including differential reporting that may be caused by external factors (stimulated reporting, known as notoriety bias, following media attention, regulatory warnings, or active pharmacovigilance projects), or related to operative choices in study design, especially the inappropriate selection of the reference group (see below).

Several reporting biases have been described, including temporal, information, selection and competition biases (Raschi et al., 2018; Faillie, 2019). Of note, the existence of biases and strategies for relevant management should be considered *a priori* during study conception and design, including a careful consideration of quality aspects such as missing data and duplicate detection (Table 1). Although data quality applies to the various SRSs, the FAERS database possesses unique features that should be carefully considered (Poluzzi et al., 2012; Giunchi et al., 2023), including unrestricted access through different public dashboards, the use of non-standardized terms (e.g., free text for doses), and most importantly, the existence of sometimes “extreme” duplicates (Hauben et al., 2007), which can inflate calculated disproportionality measures or mask the identification of other SDRs. There is no gold standard for duplicate detection (rule-based vs. probabilistic algorithm) and potential duplicates are likely to remain after cleaning procedure. Therefore, visual inspection should be considered in the case-by-case analysis (Schilder et al., 2023). Additional frequent reporting biases that can be accounted for are represented by confounding by indication (see Section 7.2) and notoriety bias, also known as stimulated reporting. If the latter is suspected, a dynamic temporal analysis of disproportionality may support proper data interpretation and avoid transforming a disproportionality signal of alert automatically into an alarm (which is not always justified) (Raschi et al., 2013).

**TABLE 1:** Major biases in disproportionality analyses and strategies for their minimization.

Type of bias	Definition	Explanation (example)	Additional notes	Minimization strategy
Data quality bias	Incompleteness (missing data) and inaccuracies of recording and codification of key fields (e.g., age, gender)	Missing data and inaccurate adverse event coding can affect the ability to detect SDR	Missing data may vary within and across databases	Quality control and de-duplication is recommended before starting the study, especially if stratified/subgroup disproportionality analyses are planned
			Missing data can limit case-by-case analysis (e.g., dose-response relationship) and the ability to carefully detect duplicates	
Notoriety bias	A form of stimulated reporting by media attention for a given drug-event association (Pariente et al., 2007)	Increased reporting frequency after regulatory warnings, or a milestone publication even if an established association is not demonstrated yet (heart failure with DPP-4 inhibitors)	It frequently occurred in the past, although latest studies have downgraded its relevance on signal detection (Hoffman et al., 2014a)	Temporal (dynamic) disproportionality or trends in reporting may help to understand the phenomenon
	A dilution effect for older drugs (when a drug class is influenced by media attention) is possible (Pariente et al., 2009)		A “ripple effect” on other drugs within the same pharmacological class is possible (e.g., rhabdomyolysis with statins after cerivastatin withdrawal)	A pre/post analysis could be considered to assess the impact
			The Weber effect (peak in reporting in the first 18 months after drug approvals) is not necessarily generalizable to all drugs (Hoffman et al., 2014b)	
Competition bias	A known drug-event combination has more chance to be notified (Arnaud et al., 2016)	Increased reporting of drug-event combinations expected for notoriety and frequency (e.g., bleeding with anticoagulants; nausea/vomiting with chemotherapy)	Also known as “masking”, since it can potentially mask the identification of rare adverse events (a form of dilution) (Maignen et al., 2014)	If suspected, sensitivity analyses are required, in which all reports related to potential competitors are excluded from the dataset (i.e., cases and non-cases)
			Drug- and event-related competition bias should be considered on *ad hoc* basis	
Co-prescription bias	Drug preferentially co-prescribed with drugs (or comorbidities) that are risk factor for the adverse event (Avillach et al., 2014)	Concomitant drugs or comorbidities are not innocent bystanders (e.g., ACE-inhibitors co-prescribed with antidiabetics and hypoglycemia)	Also called confounding by association or signal leakage	If suspected, at least one sensitivity analysis must be performed, including subgroup/stratified analysis
			It depends on pharmacokinetic and pharmacodynamic considerations	
Confounding by indication	Preferential prescription of drugs in patients at higher risk of an event	The underlying disease (diabetes) is *per se* a risk factor for the event (pancreatitis) when analyzing pancreatitis with antidiabetic drugs	It is different from the underlying diseases or treatment indications being coded as adverse events (a frequent reporting phenomenon sometimes referred to as indication bias)	Disproportionality by therapeutic area and the active-comparator restricted disproportionality analysis can be considered. Be cautious in the interpretation and search for other alternative causes
			It should be always considered when planning DA.	
Channeling bias	Differential prescription of drugs in relation to disease severity (with newer drugs usually prescribed to patients with more severe disease) (Weinstein et al., 2020)	It may be also caused by guidelines (e.g., selective prescription of SGLT2 inhibitors in subjects at high cardiovascular risk)	It should be always considered when planning DA, especially in oncology, although it was not systematically demonstrated for spontaneous reporting data	It is unlikely to be fully avoided, even after multiple adjustment models (acknowledgment among limitations)
			It may also apply to drugs within the same pharmacological class sharing the therapeutic indications but with a known different tolerability profile	

DA, disproportionality analysis; SDR, signal of disproportionate reporting.

The interpretation of negative findings and potential benefits (some beneficial drug protection) remains controversial (Antonazzo et al., 2020; Raschi et al., 2020a; Raschi et al., 2020b). Because disproportionality measures are interdependent, the lack of statistically-significant disproportionality should not be automatically interpreted as a safety endorsement: claiming that a drug is potentially free of any specific risk requires careful consideration of potential biases (e.g., masking) together with a case-by-case analysis to exclude alternative causes (see below) and consistency across other data sources; likewise, interpreting an inverse signal (i.e., a statistically-significant negative disproportionality indicating a lower-than-expected reporting) as a protective drug-related effect may be misleading (it could simply suggest a higher likelihood of underreporting for different reasons). Very recently, DAs have been provisionally explored to identify candidates for drug reposition, which have been subsequently tested *in vitro* as part of a translational approach (Chrétien et al., 2021). It is important to remark that such novel pioneering approaches should not only take into account all the potential biases that have been described so far, but also the available pharmacological evidence (Battini et al., 2023b). Any potential use of spontaneous reporting databases for drug repurposing should be possibly validated and confirmed *in vivo*, *ex vivo* and in clinical trials before becoming a consolidated approach.

## 7 Inappropriate disproportionality analysis

### 7.1 Disproportionality used as direct measure of risk

As anticipated, the presentation of results from a DA may be subject to potential misinterpretation, especially for readers and researchers not familiar with peculiarities and limitations of spontaneous reporting data. In fact, by adopting the so-called case/non-case approach (a peculiar case-control design adapted to a spontaneous reporting system), the general belief is that the higher the disproportion (e.g., ROR value), the stronger the association. As a consequence, disproportionality measures might be erroneously presented in terms of risk estimates, namely, relative risk and odds ratio (Borrelli et al., 2018).

In the recent past, different meta-research studies compared relative risks from analytical studies and meta-analyses with estimates from DAs as an aid to estimate the accuracy and performance of DAs as a possible early indicator of the likely clinical importance of an ADR (Maciá-Martínez et al., 2016; Beau-Lejdstrom et al., 2019; Gatti et al., 2021a; Khouri et al., 2021a). The observed moderate-to-weak correlations, restricted to objective type A ADRs not associated with the underlying pathology further corroborated the notion that disproportionality measures *per se* cannot be used as a proxy/surrogate of relative risk/odds ratio. Only if no data from epidemiological studies or clinical trials existed, using disproportionality measures to prioritize a given drug/event in terms of clinical importance could be acceptable (Khouri et al., 2021a), provided that appropriate biases have been accounted for (Gatti et al., 2021a; Gastaldon et al., 2022).

### 7.2 The use of restricted dataset as primary analysis

The population selected for DAs is pivotal, and every methodological choice should be justified, including pre-specified subgroup and stratified analyses (e.g., by age, sex, country) to enhance the precision of signal detection and better clarify the generalizability of results, for instance by uncovering a signal in a given subpopulation (Sandberg et al., 2020). A justified background restriction may reduce the noise in signal detection (reducing false positive while increasing true positive drug-event associations) or at least account for potential confounders. For instance, the so-called disproportionality by therapeutic area may be useful to mitigate the confounding by indication (Grundmark et al., 2014), or with the intent of including patients with similar indications through the “active-comparator restricted” DA (Alkabbani and Gamble, 2023).

However, the selection of the comparator, even if correctly chosen based on solid clinical and pharmacological considerations, may inadvertently introduce, or even enhance, the magnitude of selection bias (Gravel and Douros, 2023). For instance, if a potential cardiotoxicity for an anticancer drug emerges as a significant SDR from unrestricted primary DA, this signal could probably disappear by using all other reports in oncological therapeutic area as additional comparator, due to well-known and frequently reported cardiotoxicity associated with other oncologic drugs (resulting in a false negative signal) (Cutroneo et al., 2014). Some studies that evaluated the performance of restricted quantitative signal detection by therapeutic area for specific classes of drugs (i.e., selected anticancer therapies, orphan drugs), also confirmed that such restricted analyses, when used alone, should reduce noise but at the same time may be associated with a decrease in the sensitivity of signal detection process (Hauben et al., 2017; Sardella and Lungu, 2019).

We support, as initial (conservative) approach, the use of the full dataset (without restriction) as a first-pass signal detection approach (Cutroneo et al., 2014). This primary unrestricted analysis could be used as a benchmark for secondary/sensitivity DAs using comparators restricted *a priori* for minimizing a particular bias. These restricted analyses should be optional rather than a default analysis, especially in the oncological area (Hauben et al., 2017; Raschi et al., 2020c). The use of different (justified) comparators could be considered to assess the robustness of SDRs thus increasing relevant validity and transferability in clinical practice (Raschi et al., 2021).

### 7.3 Use of DA methods for drug-drug comparisons

Directly comparing the safety profiles of different drugs using DA is not advised. This is due to several biases inherent in spontaneous reporting data, including the lack of denominator, the presence of significant differential reporting patterns and multiple confounding factors (Michel et al., 2017).

Specifically, using DA findings as a direct measure of risk is misleading without a denominator representing drug usage and precise knowledge of the extent of under-reporting for each drug being studied. Ideally, to compare the safety data of different drugs using disproportionality measures it should be demonstrated that the drugs being studied have equal overall reporting rates and no differential reporting.

Another factor to consider is that disproportionality measures are interconnected. Variations in drug reporting patterns and significant changes in disproportionality results for specific combinations could lead to consequential shifts for other related combinations. This means, for example, that drug-AE combinations with high expected counts would need substantial increases in observed counts to yield a significant disproportionality result, and, even if they are true signals, may not emerge quantitatively. Moreover, numerous risk factors could confound apparent positive signals. Even when comparing drugs within the same therapeutic area, specific drug utilization patterns and unmeasured confounders could undermine the reliability of disproportionality findings. This underscores that direct comparisons between drugs based on DA should be approached with caution or even avoided (Greenblatt, 2015; Michel et al., 2017).

### 7.4 Use of DAs for already known drug-event associations

When conducting DAs, the definition of safety signal should always be considered. Since a signal should provide “information on a new or known adverse event that is potentially caused by a medicine and that warrants further investigation,” the conduction of DAs to describe already well-known ADRs, which are already included in the SmPC (e.g., tricyclic antidepressants-induced memory disorders), may be redundant and unnecessary o even misleading (Norén et al., 2014).

Therefore, a signal should always be characterized by elements of novelty (see paragraph 4.1). The research question of a signal detection study must therefore be directed towards the exploration of associations that are not expected due to their nature or intensity, or that are not reported in the summary of product characteristics. In the case of a known association, the research question may be directed to generate hypotheses relating to populations at higher risk than others (for example, an event may be reported for a drug more frequently in the female population than in the male population than not all other adverse reactions for that drug versus all other drugs) unless this risk has already been confirmed by clinical trials or robust observational studies. These research questions can be answered simply by a descriptive analysis of reported AEs.

In general, a signal detection study can answer to research questions aimed at confirming hypotheses supported by weak evidence such as few case reports, case series or uncontrolled studies. It is important that the notoriety of the identified signal is clearly characterized and discussed by the investigators in order to bring out the elements of novelty. This is particularly important when the analysis is conducted to investigate all possible signals of a specific drug or drug class without *a priori* hypotheses (Liu et al., 2023; Shu et al., 2023).

### 7.5 Use of disproportionality analysis when descriptive analyses would be sufficient

Some studies have performed DA using spontaneous reporting databases with the aim of describing the safety profile of a drug or a class of drugs in the real world, for instance in comparison with safety data from clinical trials (Kim et al., 2022) or in special populations (Xue et al., 2023). Providing a list of DAs without a critical insight (except sometimes for the novelty) does not provide useful information for clinicians, regulators, and other stakeholders. These research questions can be answered simply by a descriptive analysis of reported AEs. Moreover, if access to narratives is allowed, individual case assessment is useful to verify pre-existing risk factors or other subjects’ susceptibilities, thus making a cases series highly informative for clinical practice and regulatory measures such as an update of SmPC (Saely et al., 2021).

It is important to underline that the comparison between measures of disproportionality can at best highlight that one signal is stronger than another within a database, but it does not provide any comparative information on the effective risk of developing an event for a drug compared to another. Factors such as the choice of the database in which the analysis is performed and the choice of the reference for the calculation of disproportionality indices are important determinants of the strength of the signal and are independent of the actual exposure of the patients to the drugs and therefore also of the actual risks.

### 7.6 Use of DAs for adverse events with high background incidences

Although SRSs remain the cornerstone of post-marketing drug safety surveillance, judicious use of other available data sources is essential to allow a better detection, strengthening and validation of safety signals. While rare AEs that are strongly attributable to drugs (e.g., Torsades de Pointes and Stevens-Johnson Syndrome) can be adequately captured by analyses of spontaneous reporting, those that already have a high background incidence (e.g., cardiovascular events and malignancies) can be better investigated in healthcare databases (Patadia et al., 2018).

In addition, while most spontaneous reports typically involve newly marketed drugs, EHRs and claims databases data can potentially identify new risks associated with old medications (due to new indications of use or new generation of users), as well as AEs that are not easily predictable pharmacologically and less likely to be suspected as drug-induced, thus resulting in a lower likelihood of being reported. In our opinion, these systems might complement SRSs in signal detection, also for events that are not easily attributable to drugs due to their multifactorial nature or that may be coincidental (e.g., malignancies and some pregnancy outcomes).

Indeed, pharmacovigilance studies aimed at exploring the scenarios in which EHR-based signal detection systems can enhance already existing SRSs, proved that the contribution of each system to signal detection seemed to correlate with the background incidence of the events, being directly proportional to the incidence in EHR-based systems and inversely proportional in SRS. As a proof of this, it has been demonstrated that using a network of claims databases and electronic medical records could have anticipated the identification of the rofecoxib-related acute myocardial infarction signal of about 4 years before the signal was identified through SRS data (Patadia et al., 2018).

Notably, to the best of our knowledge, there are no national or regional pharmacovigilance systems that regularly and systematically use EHR or similar data to perform signal detection on events with high background rates, or at least not to the same extent as DA is used on SRS. Therefore, DA will likely remain our best option, until methods for signal detection in EHR are validated and implemented for regular use in pharmacovigilance. However, there are several ongoing initiatives that are pursuing the goal of systematically integrating the use of Real World Data to perform signal detection. For instance, the European Medicine Agency announced that with the DARWIN project the use of these data sources will part of this regulatory environment by 2025 (Arlett et al., 2022).

## 8 Communicating signal of disproportionate reporting to the right audience

Historically, published case reports of ADRs have been means to document the suspicion of occurrence, course, and management of drug-induced effects. Such works primarily carry information targeted to caregivers in specific settings; Sutherland first reported the “gray pallor” following injectable chloramphenicol in 3 neonates (SUTHERLAND, 1959), citing that their deaths were “reported in the hope of stimulating publication of the observations of others which may lead to less tentative conclusions than are possible on the basis of a few uncontrolled case reports.” These findings were confirmed in the same year by Burns et al. (1959), who noted an imbalance of ‘ashen color’ in neonates treated with chloramphenicol compared to no treatment or penicillin/streptomycin. Perhaps more famously, McBride’s letter to the Lancet asked whether readers of the journal had seen other instances of phocomelia following administration of thalidomide to pregnant women (Mcbride, 1961), culminating in the pharmacovigilance we know today. Similarly, at the time of study design, authors of DAs should keep in mind the target audience that may benefit from reading their work. Depending on the message authors of DAs may wish to deliver, regulators, researchers, caregivers, or all these stakeholders in pharmacovigilance, may be eligible audiences.

Communication to regulators can be considered when the authors’ wish is to encourage regulatory action. However, some considerations apply. For example, to the best of our knowledge, no regulatory action is solely supported by published DAs (Dias et al., 2015). Even considering the period subsequent to regulatory action, DA may not play a substantial role: the US FDA maintains a list of findings from DAs applied to FAERS, i.e., “potential signals of serious risk.” The items therein were found to mostly lead to FDA regulatory action (Dhodapkar et al., 2022). However, studies that corroborate these disproportionality signals, i.e., subsequent to and in support of the FDA communications, mainly consist of case reports or series of case reports (Dhodapkar et al., 2022). In brief, there is limited evidence on the usefulness of DAs to either support or corroborate post marketing regulatory decision-making.

When audience is represented by clinicians and other healthcare professionals, authors of DAs should be very careful, especially when drawing conclusion, since the key message may significantly impact on clinical practice, particularly if the study is (will be) published in high-ranking Journals. From a clinical viewpoint, the scientific rationale of the study and the choice of comparator are key aspects in the study design. Moreover, as anticipated, a balanced presentation and proper interpretation of key findings, notably in the abstract, is pivotal to avoid transforming an alert (i.e., a disproportionality signal) automatically into an alarm (Raschi et al., 2020c). The most important clinical implication of a published DAs is to increase awareness, thus supporting proactive monitoring and finally promoting safer prescribing.

From a research perspective, DA results can be communicated to get other researchers working on drug safety involved in the discussion and to stimulate more robust confirmatory investigations. Although the vast majority of DAs remains unnoticed, the publication of a DAs, by definition, should prompt the conduction of analytical pharmaco-epidemiological approaches (e.g., cohort studies) to confirm or refuse the association (Raschi et al., 2020d; Chan et al., 2022). Therefore, DAs should direct subsequent research, provided that a signal is considered robust and valid to be tested/verified in longitudinal healthcare databases (Harpaz et al., 2017). In this regard, the Sentinel initiative, the European Health Data Evidence Network and the Observational Health Data Sciences and Informatics program are multi-stakeholder interdisciplinary collaborative projects aiming to timely safety assessment.

As a final consideration, considering the trans-disciplinary nature of pharmacovigilance (Rocca et al., 2019), we recommend a multidisciplinary team (comprising experts in spontaneous reporting systems, clinicians, biologists, statisticians, biomedical informatics, etc.) when planning, conducting and interpreting a DA, especially in the current era of Big Data and artificial intelligence.

To enhance the validity of DAs, responsible use and publication of their results and relevant clinical transferability, the READUS-PV project (https://readus-statement.org/) is developing dedicated reporting recommendations to assist researchers, reviewers, editors, and readers in critical assessment of a manuscript and its findings, thus promoting the publication of higher-value DAs, ultimately benefiting public health.

## 9 Conclusion

The availability of large-scale SRSs has attracted considerable interest among clinicians and other stakeholders without consolidated expertise in pharmacovigilance. Of note, these archives provide public access to raw data that can be downloaded, or can be easily queried through online tools (e.g., FAERS public dashboard) by individual researchers, thus offering an unprecedented opportunity to timely and cheaply perform DAs, as demonstrated by the exponential increase in the number of publications on DAs (Wang et al., 2021; Sartori et al., 2023).

However, different meta-research studies have shown lack of transparency in reporting DAs and common misinterpretation (also called spin) of results in published studies (Khouri et al., 2021a; Khouri et al., 2021b; Cortes et al., 2023). Therefore, this review offered a perspective on methodological aspects (and understanding) of DAs, how they are carried out, reported, and could be interpreted to avoid what someone coined “apophenia” (i.e., the perception of meaningful patterns and causal connections among random data) (Gagne, 2014), or the so-called “Pharmacovigilance Syndrome” (Greenblatt, 2015), i.e., the incorrect use of spontaneous reporting systems to infer causality, calculate incidence, or provide risk stratification, which may ultimately result in unjustified alarm. A synopsis of main conceptual and technical aspects of DAs is provided in Table 2.

**TABLE 2:** Synopsis of conceptual and technical aspects in performing and reporting a DA. Modified from Raschi et al. (2018), Raschi et al. (2020c), and Raschi et al. (2023).

Study feature	Domain of clinical relevance	Comments and notes
Study concept	Rationale	Specify the scientific basis underlying the study in the introduction (see text for details)
		Justify overlapping studies (redundant publication) on the same topic, including previous pharmacoepidemiological studies
Study design	Case-by-case analysis	Clarify the intended aim (e.g., causality assessment or descriptive purpose such as demographic information and the time to onset)
		For rare events such as TdP and DILI, individual inspection of reports may be highly informative and complement a DA.
	Database	Justify the choice of spontaneous reporting system, and the time period covered by the analysis
		For some databases such as FAERS, pre-processing cleaning procedure of raw data (duplicates removal, correction of misspelled drug names) is required to ensure reliable DA.
	Case definition	Define the adverse event(s) of interest and relevant MedDRA codification (PT, SMQs, customized definition)
		Specify any restriction related to the drug role in event reporting (suspect vs. concomitant)
	Disproportionality measure(s)	Although no gold standard exists, justify the choice of disproportionality measure(s) and explicitly define relevant threshold(s) for statistical significance
	Comparator (population used as denominator)	Usually, the entire database is used as a comparator (i.e., non-cases), whereas other restrictions of the background such as disproportionality by therapeutic area should be justified by pharmacological and clinical aspects (see text for details)
		Pre-specified secondary/sensitivity analyses, including stratification and/or adjustment should be planned *a priori* to assess the consistency/robustness of findings
	Bias identification and minimization	Expected biases and measurable confounders (e.g., co-medications) should be *a priori* conceived and accounted for (see text and Table 1 for details)
Study reporting	Data presentation and interpretation	Discuss biological plausibility/pharmacological basis, and other types of published evidence (e.g., Bradford Hill criteria)
		Avoid spin (misinterpretation in presenting results): causal inference, incidence, reverse causality, safety endorsement, risk ranking (see text for details)
		Inherent caveats should be discussed among limitations

DILI, drug-induced liver injury; FAERS, food and drug administration adverse event reporting system; MedDRA, medical dictionary for regulatory activities terminology; PTs, Preferred Terms; SMQs, standardized MedDRA, queries; TdP, torsade de pointes.

Moreover, the following take-home messages can be derived from our reflections:

• Even when properly designed, conducted, and reported (taking into account limitations), we should not forget that DAs remain hypothesis-generating studies, namely, a signal detection approach, and provide the basis to carefully design prospective cohort studies and pragmatic clinical trials.
• DAs cannot be used *per se* as a standalone approach to assess a drug-related risk (they do not provide risk quantification and ranking, or safety endorsement) and cannot replace clinical judgment in the individual patient;
• Nonetheless, the role of DAs is indisputable and irreplaceable for rapid detection of rare but unpredictable AEs with strong drug-attributable component (known as designated medical event, such as TdP), especially when combined with a careful case-by-case analysis (individual inspection of reports for causality assessment or to uncover reporting patterns and clinical features), thus supporting clinicians towards proactive monitoring and safer prescribing;
• a multidisciplinary team (comprising experts in pharmacovigilance, clinicians, biologists, statisticians, biomedical informatics) is warranted to manage the various multifaceted aspects of study design and analysis (e.g., data cleaning, statistical analysis, biological plausibility).

We eagerly await the results from the international READUS-PV project (https://readus-statement.org/), which is developing specific reporting recommendations of DAs. This international collaborative effort aims to enhance transparency and support researchers in conveying the methods and results of their research, thus promoting research culture in pharmacovigilance. The project stems from already existing reporting guidelines, especially the strengthening the reporting of observational studies in epidemiology (STROBE) and the reporting of studies conducted using observational routinely collected health data statement for pharmacoepidemiology (RECORD-PE), and will also hopefully assist reviewers, editors, and readers in reproducible publication of higher-value disproportionality studies, ultimately advancing pharmacovigilance as a whole and benefiting public health. We are aware that recommendations and guidelines themselves are not sufficient to guarantee good quality of research in the absence of an appropriate research culture in Pharmacovigilance. However, these tools can support the pharmacovigilance research community in spreading this culture through continuous and constructive discussion.
